# Gleanings from Our Exchanges

**Published:** 1873-10-01

**Authors:** 


Nocturnal Incontinence of Urine cured by Circumcision.—Dr. Joseph Bell communicated to the Med. Chir. Soc., of Edinburgh, a case of nocturnal incontinence of urine which had persisted for seven years, in which he had perfomed circumcision a month previously, since which the incontinence had entirely ceased.—*Edin. Medical Journal*, May, 1873.

Radical Cure of Fistula in Ano.—Dr. Hute recommends the injection of a solution of iodine in ether instead of the ordinary tincture of iodine, on the ground that the ether, being more volatile, escapes more rapidly, leaving the walls of the fistula in contact with pure iodine. The reaction is insignificant, and there is no occasion for the patient to keep his bed.—*Le Mouvement Medicale.*

OXIDE OF ZINC IN INFANTILE DIARRHŒA.—In the *British Medical Journal* of July 12, Mr. Edward Mackey, M.B., recommends oxide of zinc in infantile diarrhœa, especially in those forms complicating nerve-troubles or hooping-cough. He points out that it has "tonic and anti-spasmodic as well as astringent qualities, a combination in a non-irritant substance exactly suited to many cases of the malady." It does not irritate, as chalk sometimes does, or gripe, as acids do occasionally, and is a better nerve-tonic than bismuth. Mr. Mackey says that it has given him excellent results in all varieties of infantile diarrhœa, in conjunction with suitable diet. Dose, one grain for any age under two years, with a little syrup, mucilage, and dill-water, three or four times daily, not on an empty stomach. A larger dose may nauseate. He says that oxide of zinc will give us in many cases the maximum of good with least liability to harm—an advantage to be desired, especially in out-patients and distant cases. Dr. Warring-Curran has referred to this use of oxide of zinc in the *Lancet*, October, 1868; and Dr. Brakenridge, in the *Medical Times and Gazette*, February, 1873, points out its tonic and anti-spasmodic as well as astringent properties.—*Philadelphia Med. Times.*

### Gleanings from Our Exchanges.

### THE BRITISH MEDICAL ASSOCIATION—ADDRESS ON SURGERY.

The following abstract of the address on Surgery, delivered at the recent meeting of the British Medical Association, by Prof. John Wood, of King's College, is from the London *Doctor* of Sept 1st:

"Prof. Wood reviewed the recent progress of Surgery, dwelling chiefly on those points on which he could speak from his own experience.

"He had the highest respect for Lister's system of treating wounds. On his theory of germs it was consistent and simple enough ; but it was as a practical method that it must be estimated; and it was with that view that he had put it as far as possible to the test. He began it when the hospital was in a good hygienic condition, and for that time the cases did admirably. He had some cases quite equal to any described by Prof. Lister. He also tried the application of dry lint, and in many, especially in breast cases, the results were also perfect. He also tried the application of the chloride of zinc solution, in the manner originated by Mr. De Morgan, and very good results ensued. After about six months a very unfavorable change came into the hospital. Erysipelas and pyæmia began to show themselves. The wounds began to suppurate more, primary healing was less common, and the erysipelatous blush appeared in cases treated in all ways, and almost as impartially on his own antiseptic side of the hospital as on his colleague Sir W. Fergusson's non-antiseptic side. But there was little or no putrefaction in any of his cases, as evidenced by the odor, which his eminent colleague, however, attributed to the carbolic acid smell overpowering all others.

"In some cases of psoas abscess treated by Lister's method, he had had marked success so long as the hospital remained healthy. But when erysipelas and pyæmia appeared, he had others, in which the pus in the abscess became putrid and offensive after the first evacuation under the spray, and with all the precautions, and he was obliged to make free openings and introduce drainage-tubes through which the abscess could be washed out with antiseptics. Such cases showed the danger of departing from the old rule of providing a free exit for all the purulent and offensive discharges, for the want of which the exclusion of air was not a sufficient compensation.

"He then alluded to Mr. Callender's paper, and the advantages of cotton-wool, which was preferred to any other dressing, he said, by Mr. Christopher Heath. He spoke of Professor Humphry's and Dr. Weil's views. He said that, after frequent trials, he considered that the readiest, lightest, coolest, and most generally useful application of the antiseptic method, was a plan comprising the free use of Chassaignac's drainage-tubes, passing from the surface of the wound, or from its interior if deep and sinuous, and with their outer extremities imbedded in cotton-wool or oakum, well permeated with MacDougall's or Calvert's powder, or other disinfectant or absorbent of discharges; the surface of the wound washed over, after bleeding had ceased, with a mixture of solutions of chloride of zinc or sulpho-carbolate of zinc; the same solution to saturate the lint, applied in the same way as in water-dressing, and enveloped in thin gutta-percha tissue, the whole supported by strapping and a light bandage. An outer envelope of cotton-wool, or oakum, completed a method from which he had obtained as good results as any, and which he had found it less difficult to ensure being carried out.

"He agreed with Mr. Lund's view. Erysipelas did not seem to be much influenced by antiseptic measures. It was a constitutional disease. Although usually showing itself first at the wounded part, it did not invariably.

"In four cases of pyæmic infection the treatment to which he had subjected his patients was attended with success. Besides persevering in the antiseptic local treatment, and giving free access of fresh air, he said he had surrounded his patients with a highly antiseptic atmosphere, by placing muslin bags of MacDougall's powder around and within the bed, and in abundance about the wound, so that he should both breathe the carbolic and sulphurous vapor, and imbibe it through the skin. If the stomach were not feeble or irritable, he also gave three to six grain doses of sulpho-carbolate, of iron with the view of testing Dr. Sansom's practice. He took care not to give the drug soon after nourishment was taken, so as not to interfere with the first stage of digestion, and discontinued it at once if the appetite fell off, or there was pain after taking it. Three of the cases showed in a week or ten days the peculiar slate-colored or olive-green color of the urine. These four cases completely recovered.

"Mr. Wood said he believed cases of recovery frequently occurred under other methods or no methods, and that as much depended on the age and reparative power of the patient, the amount of blood-poison formed or absorbed, and the general conditions of the atmosphere, as upon any system of treatment whatever. He attached much importance to free drainage in dressing wounds. When these were made by the surgeon a good deal might be done by a judicious choice of the direction of the incision in resections, etc., position of flaps, etc., in amputations. Mr. Hutchinson's plan of making a puncture in the popliteal space, in excision of the knee-joint, illustrated his meaning. The wound should be made to slope towards that part which was most dependent when the patient was laid in bed. For that reason the circular operation in amputations of the thigh was objectionable. Very good drainage was accomplished in the late Mr. Teale's plan of a single square anterior flap. He preferred in the thigh, however, an oblique double flap, with the outer end of the incision placed lower than the inner, and the front flap placed somewhat outside the limb, and longer than the hinder. He was convinced that this gave the most complete drainage, prevented the bone protruding, and made a shapely and serviceable stump, with the cicatrix placed well behind the point of pressure.

"Another important point in favoring the escape of discharges from the interior of a wound, lay in the manner of securing the arteries. He believed, with Sir W. Fergusson, that so long as entire union was wanting, ligature threads might have the advantage of keeping open channels for the escape of discharges from the close neighborhood of the tied arteries, the accompanying veins of which were frequently the source of effusious of blood after the wound was dressed, and these would afterwards clot and putrefy. He usually well steeped the ligature threads in carbolized oil, saturated so as to be unable to absorb discharges, and utilized so as to spread around an antiseptic influence. There was one point in the section of flaps which he thought might have influence sometimes on the introduction of pus or septic matter into the cut veins. When these were cut obliquely with the face of the flap, they were opened in a large conic section in the shape of a pen, and left, when placed on the underlying flap, in an attitude well adapted for receiving and conducting into their interior pus and putrid discharges which gravitated from the surrounding hollow and often funnel-shaped sides. To obviate this, he invariably, after a flap operation, cut off the larger veins transversely.

"Mr. Wood next touched upon the radical cure of rupture. He said he had long thought we might prevent a return of the protusion after the operation for the relief of strangulation. He believed that, in a healthy subject, the peritoneum might be dealt with as freely and as safely as any tissue; and that the chances of bad results from peritonitis would depend upon the injury sustained by the bowel in peritonitis, rather than upon any way of dealing with the peritoneal sac and parietes, after the strangulation had been relieved, provided due drainage were secured. When the bowel and omentum were congested only, and most likely to recover when placed in their natural cavity, especially in young and healthy subjects, he concluded that the attempt would be justified, and probably be successful. The advantage of preventing a life-long trouble was obvious. He then narrated three cases.

"Mr. Wood said, cases of ectopia vesicæ and epispadias possessed an interest for surgeons who had a tendency to plastic efforts. During the last ten years that his attention had been directed to this deformity, he had met with forty or fifty cases. More had been recorded by Prof. T. Holmes, who first practiced successfully in this country, in 1863, the plan followed by Paucrost and Ayres in America in 1858. It had apparently been suggested by M. Richard's unsuccessful application of Nelaton's principle of laying a flap upon the parts to be covered, with the skin surface downwards, and covering it with another. Other cases had been operated on in various ways by Messrs. Simon, Lloyd, Sydney Jones, and Thos. Smith, with, he believed, little success. He had himself operated upon sixteen cases.

"For epispadias, he had operated by Nelaton's method twice, with favorable results. For hypospadias, in which the urethra was deficient below as far as the scrotum, he had operated three times upon a plan somewhat similar, viz.: a reversed flap upon the side of the opening, with another from the scrotum superimposed upon it.

"A similar plan, combined with internal section and dilatation of the strictured urethra, he had found to succeed admirably and completely in two cases of penile urinary fistula communicating with the urethra in front of and close to the scrotum, accompanied with a hard cartigalinous stricture of the urethra.

"Among the novelties of the year was the plan of M. Demarquay, of passing a long flexible hollow bougie through a perineal urinary fistula previously enlarged by the bistoury, and then one end forwards through the stricture and out at the meatus, and the other end backwards into the bladder.

"Mr. Furneaux Jordan's plan of treating obstinate impassable strictures by attacking them in the rear, through an opening from the rectum into the dilated urethra behind the stricture, was a valuable contribution to surgery.

"Much had been done in other kinds of plastic surgery of late years. Since his own successful case of transplantation of skin from the abdomen to the arm, for the relief of a frightful deformity of the wrist (published in the "Medico-Chirurgical Transactions for 1863," vol. xlvi.), he had had two more successful cases. A similar operation had been also successfully performed by Mr. Thomas Smith, in St. Bartholomew's Hospital; and his colleague, Mr. Hy. Smith, had lately had a very good case of operation, upon the same plan, for the relief of an extreme contraction of the elbow. In rhinoplasty, in which so much good work had been done by Mr. Hamilton, of Dublin, he had had some good cases of restoration of the nose from the cheeks and upper lip, some of which were found among the photographs laid before the meeting. The last case he had undertaken was about the most difficult one he had ever had to deal with, in the width of the chasm and the extensive loss of substance. The nasal bones, the vomer, ascending process, and a considerable part of the alveolus of the superior maxillary, and the whole of the ethmoid, except the cribriform plate, had necrosed away, as the result of scrofulous lupus. A great prominence of the frontal sinus also constituted a source of difficulty. To afford a basis for the new nose to be turned down from the forehead, he had resorted to the plan frequently followed successfully by Sir William Fergusson, of dissecting off and bringing towards the median line both cheeks. To secure the union of these, and to efford a prominence to the point of the nose, he had followed a plan which he had before employed in bad cases with satisfactory results. At the same operation he turned up the middle third of the upper lip and split it from its lower edge, turning over the borders so as to afford a bridge of support, and an extended raw surface for union with the opposed cheeks. This was rendered necessary to enable the cheeks to meet in the median line, as well as to insure their union to each other. When this had entirely healed, by a second operation he dissected up the mucous membrane from the bridge made of lip tissue, and reflected it over the gap still left between the eyes, so as to form another bridge of support for the root of the new nose, and to prevent it from sinking into the gap so as to become too much depressed. He then turned down the new nose from the forehead, after the Indian method, fitting it on to the raw surface, which gave it admirable support, and kept up the point of the new nose in a manner which one does not commonly see after this operation.

"He had practiced with advantage M. Reverdin's method in many rhinoplastic cases to eke out any deficiencies of skin in corners where it was desirable to prevent puckering from contraction, or to substitute skin tissue for mucous membrane exposed by reversal of the upper lip structure, which was apt to show its parentage unless subjected to more friction than was agreeable or possible on a newly-formed nose.

"Mr. Wood said he had barely time, among the recent innovations of modern surgery, to notice one which "*l'audace*" of the French school is applying in a manner which those who have reached the rest-and-be-thankful stage in surgery might call daring, if not rash. He alluded to the use of M. Dieulafoy's *aspirateur* to relieve distension by puncture of the bladder in cases of retention of urine, and of the intestine in abdominal obstruction, and in joints affected with hydrops articuli, and even to evacuate the fluid contents in strangulated hernia. In the first-named of these conditions, it was no doubt valuable as an adjunct to other remedies. Demarquay had not found it of much practical use in intestinal obstruction, because of the rapid re-formation of flatus, which corresponded to his own experience of puncture in desperate cases without the aspirator. Mr. Wood said he had used it in the punture of joints and the cure of hydatids, though not frequently enough to pronounce upon its capabilities; but he had not as yet arrived at the measure of audacity to use it in strangulated hernia, as it scarcely agreed with his own impression as to the importance of avoiding any injury to the bowel in dealing with strangulated or other herniæ."

The Cases of the Three Sisters in whom the Uterus and Ovaries were Absent.—In all three subjects the vagina was short, and ended in a cul de sac; the mammæ were developed; and the marks of puberty generally existed, except that they had not menstruated. Their ages were sixteen, eighteen, and twenty-six years. No trace of uterus or ovaries could be felt. Dr. Phillips reported the cases of two sisters. Dr. Rogers had examined three similarly affected subjects. The general opinion prevailed that the ovaries existed in all such cases (although they could not be felt), as there were the marks of womanhood in the general tastes and feelings of the cases, contour of body, and sexual desires.

Many instances are upon record of this peculiar malformation affecting two or more members of the same family, as there are also of other sexual deformities, such as hypospadias, etc.— *Western Lancet.*

